# Scoliosis with hyperkyphosis combines in 20% of cases with Scheuermann's disease, and is more frequent in males

**DOI:** 10.1186/1748-7161-8-S1-O3

**Published:** 2013-06-03

**Authors:** S Atanasio, S Donzelli, F Zaina, A Negrini, S Negrini

**Affiliations:** 1 ISICO (Italian Scientific Spine Institute), Milan, Italy; 2 University of Brescia, Italy; 3 IRCCS Don Gnocchi Milan, Italy

## Background

For most scoliosis cases, the etiology is uncertain. It's possible, however, that a certain number of idiopathic scoliosis cases could be classified by known vertebral spine pathologies. It's been described in the literature that idiopathic scoliosis is related to dorsal hypokyphosis, but the existence of scoliosis in which this doesn't happen has been proved. Scheuermann's (SCH) disease, in addition to hyperkyphosis, sometimes presents also average severity scoliosis. We wondered in how many cases a SCH disease, with a prevailing lateral component, would cause important scoliosis in association with hyperkyphosis.

## Aim

To verify the presence of vertebral alterations connected to Scheuermann's disease in scoliosis considered idiopathic.

## Methods

Design: Cross-sectional study. Population: inclusion criteria was principal diagnosis of Idiopathic Scoliosis with a curve of at least 20° Cobb; sum of the sagittal plane between C7 and L3 of 90 mm. From a database of 2,432 patients affected by scoliosis greater than 20°, 201 subjects (49 male and 152 female) satisfied those conditions. All the patient's radiographs (769 overall) have been evaluated in order to identify the presence of the signs of Scheuermann disease in one vertebra at least.

## Results

Scoliosis with combined increase of the curves on the sagittal plane is 8.3%. In this subgroup, males are 24.4%. Radiographic signs of Scheuermann's disease have been found in 45 patients (23.3% of the 201 subjects under study). Among those, 14 were male (31.1%) and 31 female. Comparing to the classification according to Lenke, 6 of them were of type 1, 31 of type 3, and 8 of type 5.

## Conclusion

Among IS with combined hyperkyphosis, the prevalence of the male sex is greater than what is reported for the common form of IS. According to our results, in a significant number of idiopathic scoliosis, alterations related to Scheuermann’s disease have been reported in one or more vertebrae: in these cases the rate of males slightly increases. This could imply the exclusion of this subgroup from IS.
